# Neurosteroids and translocator protein 18 kDa (TSPO) ligands as novel treatment options in depression

**DOI:** 10.1007/s00406-024-01843-7

**Published:** 2024-07-08

**Authors:** Marco Riebel, Lisa-Marie Brunner, Caroline Nothdurfter, Simon Wein, Jens Schwarzbach, Philippe Liere, Michael Schumacher, Rainer Rupprecht

**Affiliations:** 1 Department of Psychiatry and Psychotherapy, University Regensburg, Universitätsstrasse 84, 93053 Regensburg, Germany; 2 U1195 Inserm and University Paris-Saclay, Le Kremlin-Bicêtre, Paris, 94276 France

**Keywords:** Neurosteroids, TSPO, Depression, Treatment, GABA_A_ receptor

## Abstract

Recently, the gamma-aminobutyric acid (GABA) system has come into focus for the treatment of anxiety, postpartum depression, and major depressive disorder. Endogenous 3α-reduced steroids such as allopregnanolone are potent positive allosteric modulators of GABA_A_ receptors and have been known for decades. Current industry developments and first approvals by the U.S. food and drug administration (FDA) for the treatment of postpartum depression with exogenous analogues of these steroids represent a major step forward in the field. 3α-reduced steroids target both synaptic and extrasynaptic GABA_A_ receptors, unlike benzodiazepines, which bind to synaptic receptors. The first FDA-approved 3α-reduced steroid for postpartum depression is brexanolone, an intravenous formulation of allopregnanolone. It has been shown to provide rapid relief of depressive symptoms. An orally available 3α-reduced steroid is zuranolone, which also received FDA approval in 2023 for the treatment of postpartum depression. Although a number of studies have been conducted, the efficacy data were not sufficient to achieve approval of zuranolone in major depressive disorder by the FDA in 2023. The most prominent side effects of these 3α-reduced steroids are somnolence, dizziness and headache. In addition to the issue of efficacy, it should be noted that current data limit the use of these compounds to two weeks. An alternative to exogenous 3α-reduced steroids may be the use of substances that induce endogenous neurosteroidogenesis, such as the translocator protein 18 kDa (TSPO) ligand etifoxine. TSPO has been extensively studied for its role in steroidogenesis, in addition to other functions such as anti-inflammatory and neuroregenerative properties. Currently, etifoxine is the only clinically available TSPO ligand in France for the treatment of anxiety disorders. Studies are underway to evaluate its antidepressant potential. Hopefully, neurosteroid research will lead to the development of fast-acting antidepressants.

## Introduction

Major depressive disorder (MDD) is a serious condition with a high 12-month prevalence of about 6% and a lifetime incidence of about 16% [1]. Unmet needs include the limited efficacy of antidepressants, particularly their slow onset of action. Most antidepressants commonly described in depression are believed to compensate for monoaminergic and anticholinergic neurotransmitter shifts [2], but it takes several weeks for a clinically meaningful onset of action to occur. Thus, there is a need for rapid-acting antidepressants using novel pharmacologic targets. For example, ketamine has been shown to induce relatively rapid but short-lasting improvement in depressive symptoms via the glutamate system [3]. Benzodiazepines are comparably fast-acting and widely used anxiolytic compounds [3–6]. However, side effects such as tolerance development and abuse liability limit their use for medium- and long-term treatment [3–6]. Benzodiazepines do not have sustained antidepressant properties [7–10]. Therefore, other treatment options, that produce rapid antidepressant and/or anxiolytic effects without the side effects of benzodiazepines are needed for the treatment of affective disorders. Most of the available antidepressants are based on the concept of monoamine reuptake inhibition, e.g., selective serotonin reuptake inhibitor (SSRIs). Recently, however, the GABAergic system has come into focus with the industrial development of GABAergic steroids as novel treatment options for postpartum depression and major depressive disorder. Moreover, given the discrete signs of neuroinflammation in depression as revealed by positron emission tomography (PET) studies for the translocator protein 18 kDa (TSPO) and the ability of TSPO ligands to promote endogenous steroidogenesis [5, 11], TSPO ligands may represent an interesting alternative to the administration of exogenous steroids.

Therefore, we discuss below recent developments in the treatment of depression and postpartum depression using GABAergic steroids in relation to their mechanism of action and in relation to TSPO ligands.

### Steroids: synthesis and mechanisms of action

**Fig. 1:**
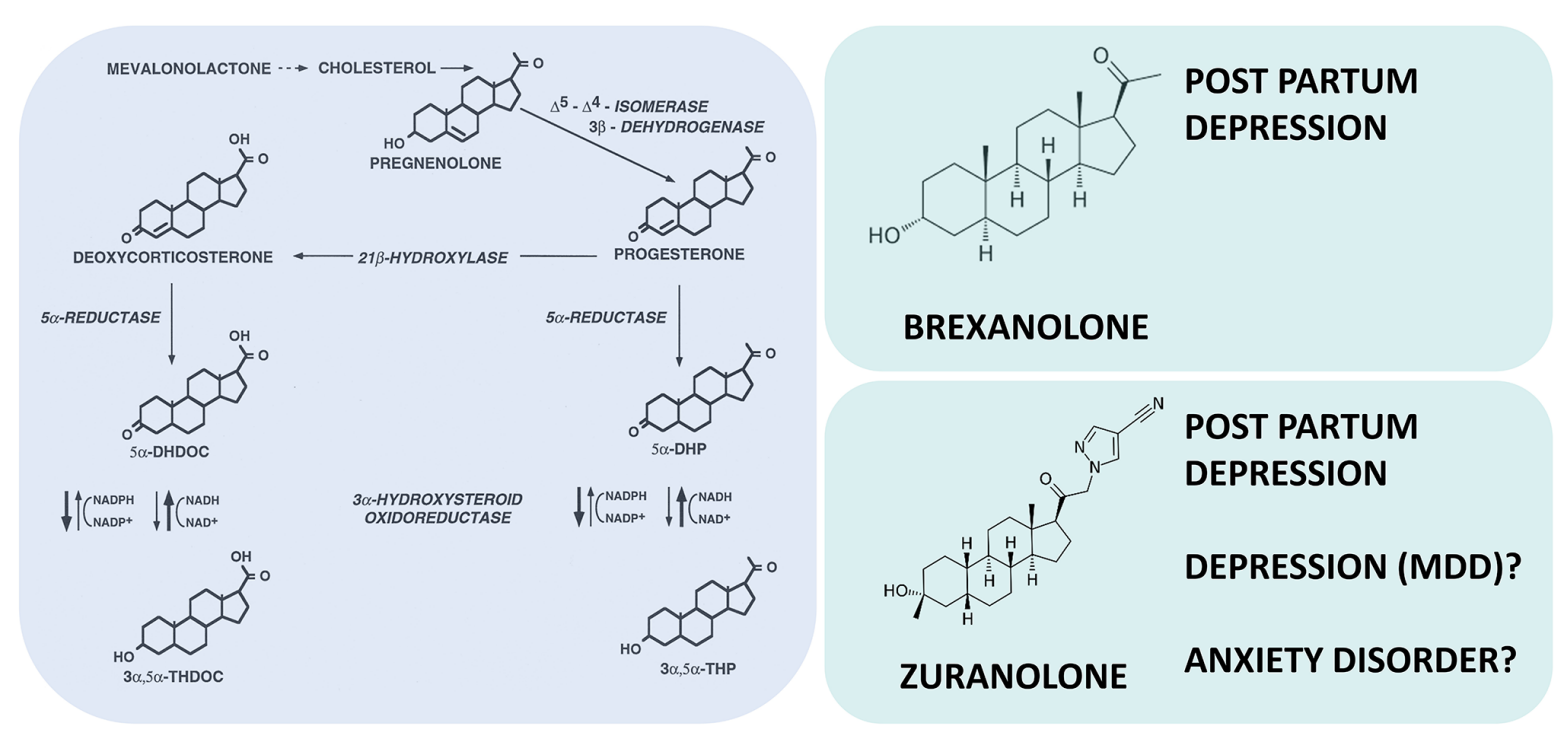
Neurosteroidogenic pathway and therapeutic compounds. Left panel: 3α-reduced steroids are metabolites of progesterone. Their synthesis is catalyzed by the 5α-reductase and the 3α-hydroxysteroid oxidoreductase. The activity of the 3α-hydroxysteroid oxidoreductase may work both towards reduction and oxidation [92]. Right panel: Molecule structure of brexanolone and zuranolone. Brexanolone is an intravenous formulation of 3α, 5α-tetrahydroprogesterone (3α, 5α-THP, allopregnanolone). Zuranolone is a synthetic modification of allopregnanolone and can be administered orally [11, 38, 43]. Brexanolone and zuranolone are approved by FDA for the treatment of postpartum depression, while zuranolone is still under investigation for major depressive disorder (MDD) and eventually for anxiety disorders

Steroids present in the brain originate from both steroidogenic tissues, e.g., the adrenal cortex and also from a local synthesis [12]. Cholesterol is a substrate for the translocator protein 18 kDa (TSPO), which serves as a channel to transport cholesterol into the mitochondrial matrix [13, 14]. There, steroids are formed from cholesterol (Fig. 1).

**Fig. 2:**
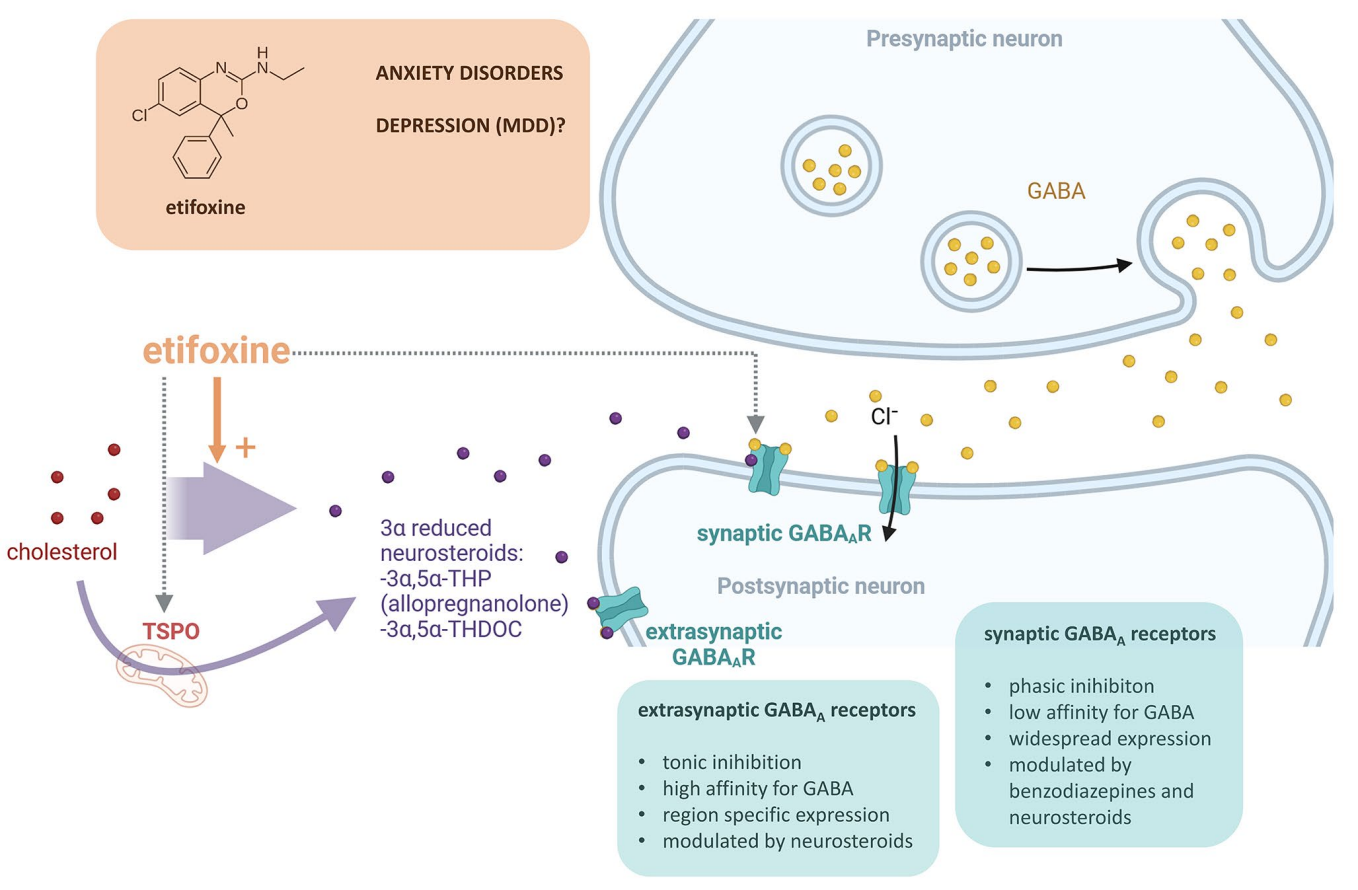
Mechanism of action of etifoxine. Upper box: Molecule structure of etifoxine. Etifoxine has a dual mode of action. It is a TSPO ligand and promotes neurosteroidogenesis, which in turn modulate GABA_A_ receptors, but also directly modulates α2 and α3 containing GABA_A_ receptors (gray dotted arrows). It is approved in France for adjustment anxiety disorders and under investigation for the treatment of depression by our group. Lower boxes: GABA_A_ receptors are expressed both at synaptic and extrasynaptic sites with different expression patterns and physiological and pharmacological properties. While benzodiazepines target only synaptic receptors, neurosteroids modulate both synaptic and extrasynaptic receptors, which may explain their distinct clinical profile

An initial steroid is pregnenolone, which is converted to progesterone and deoxycorticosterone. These steroids are further reduced via the 5α-reductase and then by the 3α-hydroxysteroid oxidoreductase that works in both directions [12] to produce 3α-reduced steroids, such as allopregnanolone and 3α,5α-tetrahydrodeoxycorticosterone (3α5α-THDOC), that are positive allosteric modulators of GABA_A_ receptors. Steroid binding occurs mainly at ß-subunit interfaces [15], whereas the benzodiazepine binding site is determined by the differential composition of α-subunits [16], thereby enhancing GABA-gated chloride currents. GABA_A_ receptors can be differentiated into synaptic and extrasynaptic GABA_A_ receptors. Synaptic GABA_A_ receptors, which are targeted by benzodiazepines and 3α-reduced steroids, show a widespread expression profile and mediate a phasic inhibition. Extrasynaptic GABA_A_ receptors, however, are targeted by 3α-reduced steroids and confer tonic inhibition with a more region-specific expression profile [17] (Fig. 2). As 3α-reduced steroids target both synaptic and extrasynaptic GABA_A_ receptors [18–20], this may contribute to their unique pharmacological profile. It is also noteworthy that while benzodiazepines prolong the fast phasic postsynaptic response of γ2 subunit-containing GABA_A_ receptors, 3α-reduced steroids additionally evoke a tonic persistent inhibitory response involving extrasynaptic GABA_A_ receptors containing the δ subunit [21]. These differential effects on the time course and peak amplitude of GABA-evoked chloride currents may explain the differences in efficacy and side effect profile between 3α-reduced steroids and benzodiazepines.

### Neurosteroids in depression

Studies investigating the composition of neurosteroids in depressed patients are rather rare. First clinical studies reported reduced levels of 3α-reduced steroids in corticospinal fluid and plasma of depressed patients and a subsequent increase following SSRI treatment [22–24]. These findings are in line with molecular data showing that SSRIs or mirtazapine may increase concentrations of 3α-reduced steroids through shifting the activity of the 3α-hydroxysteroid oxidoreductase towards the reductive direction (Fig. 1) [25, 26]. This mechanism may contribute to the antidepressant and anxiolytic effects of these compounds. Moreover, following medication with different antidepressants we found an increase in 3β-reduced steroids, which exert functional antagonistic properties [22–24]. To investigate whether these changes in neurosteroid composition more likely reflect clinical response patterns, we did a series of studies with nonpharmacological treatments of depression such as sleep deprivation [27], repetitive transcranial magnetic stimulation [28] and electroconvulsive therapy [29]. However, none of these treatments affected neurosteroid composition independent of treatment response. On the other hand, treatment with mirtazapine normalized the altered neurosteroid pattern in both responders and non-responders to treatment, suggesting a pharmacological effect via neurosteroidogenic enzymes [26]. Another study suggested alterations in neurosteroid composition also in perinatal depression [30]. Interestingly, in patients with premenstrual dysphoric disorder (PMDD), the sensitivity of exogenously administered allopregnanolone was shown to be dependent on the menstrual cycle [31]. Although 3α-reduced steroids are altered in depression, these changes are far less pronounced than in postpartum depression and conflicting results have been reported on the relationship between hormonal changes and symptom onset [32]. Although the reported findings of alterations in neurosteroid composition are subtle and only few studies are available, there is a good rationale for the therapeutic use of either exogenous or endogenous neurosteroids in affective disorders, which may provide a basis for novel treatment options.

### Therapeutic effects of 3α-reduced steroids

Although 3α-reduced steroids and their behavioral properties have been known for decades, only recently industrial efforts have been undertaken to put these molecules forward as therapeutic agents. The first neurosteroid to receive approval for the treatment of postpartum depression by the FDA is brexanolone [33]. The formula of brexanolone is identical to that of the naturally occurring allopregnanolone (Fig. 1). It is a special preparation for intravenous application, that should be administered for 60 h [34, 35]. In these two studies, a rapid, clinically meaningful antidepressant effect was observed that persisted throughout the study period of 30 days. The most common adverse effects were dizziness and headache. These findings were replicated in the HUMMINGBIRD clinical program with rapid improvement of depressive, anxiety and insomnia symptoms [36]. A post-marketing survey revealed no serious safety concerns for brexanolone, with sedation being the most serious side effect [37]. From a pathophysiological perspective the administration of 3α-reduced steroids such as brexanolone may alleviate affective symptoms in postpartum depression [11]. After parturition, progesterone and its 3α-reduced metabolites show an enormous decline, which may cause affective symptoms and may be alleviated by exogenous administration of 3α-reduced steroids [5]. Thus, neurosteroid replacement may be directly related to the pathophysiology of postpartum depression. However, a practical inconvenience of brexanolone is the 60-hour intravenous infusion. An oral medication was needed and was introduced with zuranolone (Fig. 1), which solves the problem of administration. In 2023, zuranolone was approved by the FDA for the treatment of postpartum depression [38, 39]. It was shown that zuranolone produced a reduction in depressive symptoms within 15 days of treatment that lasted until day 45 [39, 40]. In addition, zuranolone improved sleep patterns in a model of insomnia in healthy volunteers [11, 41]. In these studies, side effects of zuranolone were similar to those of brexanolone (headache, dizziness, and somnolence). The approval of two 3a-reduced steroids for postpartum depression represents a major breakthrough in the treatment of this mental disorder.

A series of studies have been conducted to further study the efficacy of zuranolone in major depressive disorder. Zuranolone significantly improved depressive symptoms within 15 days of treatment when compared to placebo [42] and increased patient-reported quality of life within this time frame [43]. In addition, a recent meta-analysis pointed towards a beneficial effect of zuranolone within two weeks of treatment [44]. Several follow-up studies have been published recently. A study with 50 mg zuranolone was able to reproduce the superiority over placebo on day 15 in reducing the Hamilton Depression Scale (HAM-D) score with the first signs of onset already on day 3 [45]. However, in the so-called MOUNTAIN study, zuranolone did not meet its primary endpoint at a dose of 30 mg on day 15 of treatment [46]. In the SHORELINE study, an open-label phase 3 trial, repeated treatment courses were offered as needed, with responders requiring fewer than two treatment courses during one year of follow-up [47]. Another recent meta-analysis suggested antidepressant and anxiolytic efficacy of zuranolone with an optimal dose of 30 mg, with an increased risk of side effects with increasing the dose [48]. It is noteworthy, however, that in contrast to postpartum depression, zuranolone has not been approved for major depressive disorder. Concerns have been raised about limited efficacy, incompatibility with breastfeeding, putative impairment of psychomotor function, and potential for abuse [49]. Other issues that need to be evaluated include how long the effects of zuranolone can be maintained after treatment is discontinued, whether zuranolone can really work as an interval therapy requiring only short treatment intervals of 14 days, thereby changing the course of the disease without the need for maintenance or relapse medications, and whether zuranolone can be administered for a longer period of time in relation to side effects. So far, headache, somnolence and dizziness are the most prominent side effects within a two-week treatment. The side effect profile over longer treatment periods remains to be determined. It will be of great interest to see what the future potential of zuranolone in the treatment of major depressive disorder will be.

### Structure and function of TSPO

An alternative to the administration of exogenous 3a-reduced steroids is the promotion of endogenous neurosteroidogenesis. Much research has focused on the translocator protein 18 kDa (TSPO), suggesting it as a promising candidate for endogenous steroid formation. The following section on the structure and function of TSPO is taken in part from a recent review by our group in the same journal and presented again here for reasons of clarity [11]. The translocator protein 18 kDa (TSPO) is a 169 amino acid comprising protein of the outer mitochondrial membrane (OMM) [13, 14], which is associated with other proteins residing in the OMM such as voltage-dependent anion channel (VDAC), but also with cytosolic proteins, e.g., the steroidogenic acute regulatory protein (StAR) and proteins of the inner mitochondrial membrane (IMM), such as the adenine nucleotide transporter (ANT) [13, 14]. TSPO mediates numerous biological functions such as mitochondrial cholesterol transport, porphyrin transport and heme synthesis, apoptosis, cell proliferation, and transport of ions and metabolites [13, 14]. Although TSPO is particularly abundant in steroid producing tissues it can be found substantially also in the brain, liver, heart, and the immune system. The broad range of expression and its pleiotropic functional properties render TSPO an interesting target for many disease areas [13, 14, 50]. Currently, the exact role of TSPO for steroidogenesis has been challenged by studies in various knock-out mice [51–53], where steroidogenesis remains unaffected by TSPO knockout.

Nevertheless, TSPO ligands exert numerous functions including anti-inflammatory properties, which on the one hand offer a much broader pharmacological profile but on the other hand should be considered when developing TSPO ligands for therapeutic purposes both in terms of clinical and side effects. Recently, some TSPO ligands, such as the benzodiazepine diazepam (which also has some affinity for TSPO [14]), have been shown to activate synaptic pruning, leading to increased synaptic C1q deposition, removal of excitatory synapses, microglial phagocytosis of synaptic proteins, and decline in cognitive function [5, 54]. Such findings of impaired cognitive function, which may be due to a loss of hippocampal and cortical excitatory synapses, may provide an explanation for why certain benzodiazepines, such as diazepam, may cause cognitive impairment in humans. On the other hand, a recent study showed that the TSPO ligand XBD173 prevented the amyloid β-induced neurotoxicity and dendritic spine loss and even exerted precognitive effects in a mouse model of Alzheimer’s disease [55]. Thus, the question of whether TSPO ligands may have beneficial or even detrimental effects on cognition and in neurodegenerative disorders warrants further investigation.

### TSPO ligands in the treatment of anxiety and depression

TSPO expression and activity [11, 13, 14, 56, 57] play an important role for psychiatric disorders and their treatment. A variety of studies have investigated the expression of TSPO in stress-related disorders [5, 11, 50]. These studies investigated either the expression of TSPO mRNA in peripheral mononuclear cells, the binding characteristics of the TSPO ligand PK11195 to platelet membranes, or protein expression in thrombocytes [58–60]. Meanwhile, various PET studies reported increased TSPO expression in depression [61, 62] and obsessive-compulsive disorder [63]. Recently, it has been suggested that TSPO PET imaging may even predict the clinical response to celecoxib treatment in major depression [64], thereby highlighting the potential of TSPO ligands as personalized approaches both in diagnostics and for selecting treatment procedures in relation to their outcome. In patients suffering from PTSD, it has been shown that higher C-reactive protein (CRP) levels are associated with lower prefrontal-limbic TSPO availability and PTSD severity [65]. Moreover, in neurodegenerative disorders, such as Alzheimer´s disease but also in depression with cognitive impairment, upregulation of TSPO labeling has been reported in PET studies [62, 66]. However, TSPO expression should not unequivocally be considered as a marker of neuroinflammation, since also neuronal activation may increase TSPO levels [67]. These findings suggest that TSPO ligands exert antidepressant effects through their anti-inflammatory properties. Moreover, TSPO gene variants, such as the rs6971 Ala147Thr polymorphism, which affects ligand binding and cholesterol uptake, should be considered in clinical studies when assessing TSPO binding or function. For example, both bipolar disorder and diurnal cortisol rhythm in bipolar disorder have been linked to this TSPO polymorphism [68, 69].

Various TSPO ligands may display anxiolytic or putative antidepressant properties in rodents [11, 14, 70]. In a translational study, our group showed that the selective TSPO ligand XBD173 enhanced GABAergic neurotransmission in brain slices via the induction of neurosteroidogenesis and effectively reduced the number of pharmacologically induced panic attacks in rodents in the absence of sedation [70]. Moreover, XBD173 displayed anti-panic and anxiolytic efficacy in humans using an experimental anxiety paradigm involving a challenge with cholecystokinin tetrapeptide (CCK-4). Whereas the benzodiazepine alprazolam caused sedation and withdrawal symptoms after only 7 days of treatment, these were absent in the XBD173-treated subjects. Recent molecular studies using global or neuronal TSPO knockout mice further dissected the role of neuronal TSPO for modulating anxiety- and depression-related behavior and the effects of XBD173 [57]. A recent animal study suggested that the TSPO ligand AC-5216 (XBD173) may exert rapid antidepressant and memory enhancing effects [71]. The capability of TSPO ligands to exert anxiolytic and antidepressant effects has also been demonstrated for other novel TSPO ligands such as YL-IPAo8 in a rat model of postpartum depression [72] or the antagonistic ligand ONO-2952 [73]. Therefore, TSPO represents a promising target for the development of fast-acting anxiolytics and antidepressants with a favorable side effect profile.

Currently, the only clinically available TSPO ligand is etifoxine (Fig. 2), which is approved in France. Etifoxine has a dual mode of action, as it targets TSPO but also directly α2 and α3 containing GABA_A_ receptors [74]. Initial clinical studies with etifoxine have provided first evidence for a clinical anxiolytic effect, which showed comparable efficacy to the benzodiazepine lorazepam in patients suffering from adjustment disorders with anxiety [75]. The anxiolytic effects of etifoxine comparable to clonazepam have recently been confirmed in a randomized controlled double-blind clinical trial in patients with anxiety disorders [76]. At the cellular level, we have characterized the effects of etifoxine in comparison to benzodiazepines regarding neurosteroidogenesis [56]. Moreover, our group recently described differential effects of etifoxine and alprazolam on hypothalamic-pituitary-adrenal (HPA) axis activity in the Trier Social Stress Test in Virtual Reality (VR-TSST) [77]. Furthermore, we recently showed that etifoxine but not alprazolam reduced the fear-potentiated startle in comparison to placebo in an experimental threat paradigm in healthy volunteers [78]. However, the brain circuits underlying the anxiolytic and/or anti-stress effects of TSPO ligands have not been identified so far. Therefore, we conducted a double blinded within-design study with healthy male volunteers to address this issue. In this study, we could also show differential effects of etifoxine and alprazolam on GABAergic function as assessed by double-pulse transcranial magnetic stimulation (TMS) [79] and subtle effects on microbiome composition [80]. To address the question of whether etifoxine may affect peripheral steroid concentrations, steroid profiles were obtained in plasma in a subset of study participants which are reported below.

### Steroid plasma profile following treatment with etifoxine

The study population of 36 male participants between the ages of 18 and 55 years was screened by a physician for the absence of physical and psychiatric disorders by physical examination and the German version of the Mini-International Neuropsychiatric Interview (M.I.N.I.) [81, 82]. Study population and design are described in more detail elsewhere [79, 80]. The trial was registered at the European Clinical Trials Register (EudraCT number: 2018-002181-40) and at the German Register of Clinical Studies (DRKS-ID: DRKS00020267) and approved by the ethics committee of University of Regensburg and the German Federal Institute for Drugs and Medical Devices (BfArM). All participants gave their written informed consent. Throughout the experiment, participants were required to abstain from alcohol, driving, and the use of heavy machinery. The order of medication intake was pseudo-randomly assigned - placebo, alprazolam (1.5 mg/d in 3 doses of 0.5 mg) and etifoxine (150 mg/d in 3 doses of 50 mg) for 5 days each, with a washout period of at least 7 days between medications. Due to the complexity and expense of the steroid analysis, only placebo and etifoxine samples from 25 subjects were complete and could be analyzed. These were collected on day 5, 60 min after the 12:00 midday medication intake (placebo or etifoxine). Steroid profiles were determined by means of a highly specific and sensitive gas chromatography coupled to tandem mass spectrometry analysis as described previously [53]. A two-sided t-test was performed between etifoxine and placebo samples per neurosteroid using a bootstrapped (k = 10,000) null distribution generated from the study data.

**Fig. 3:**
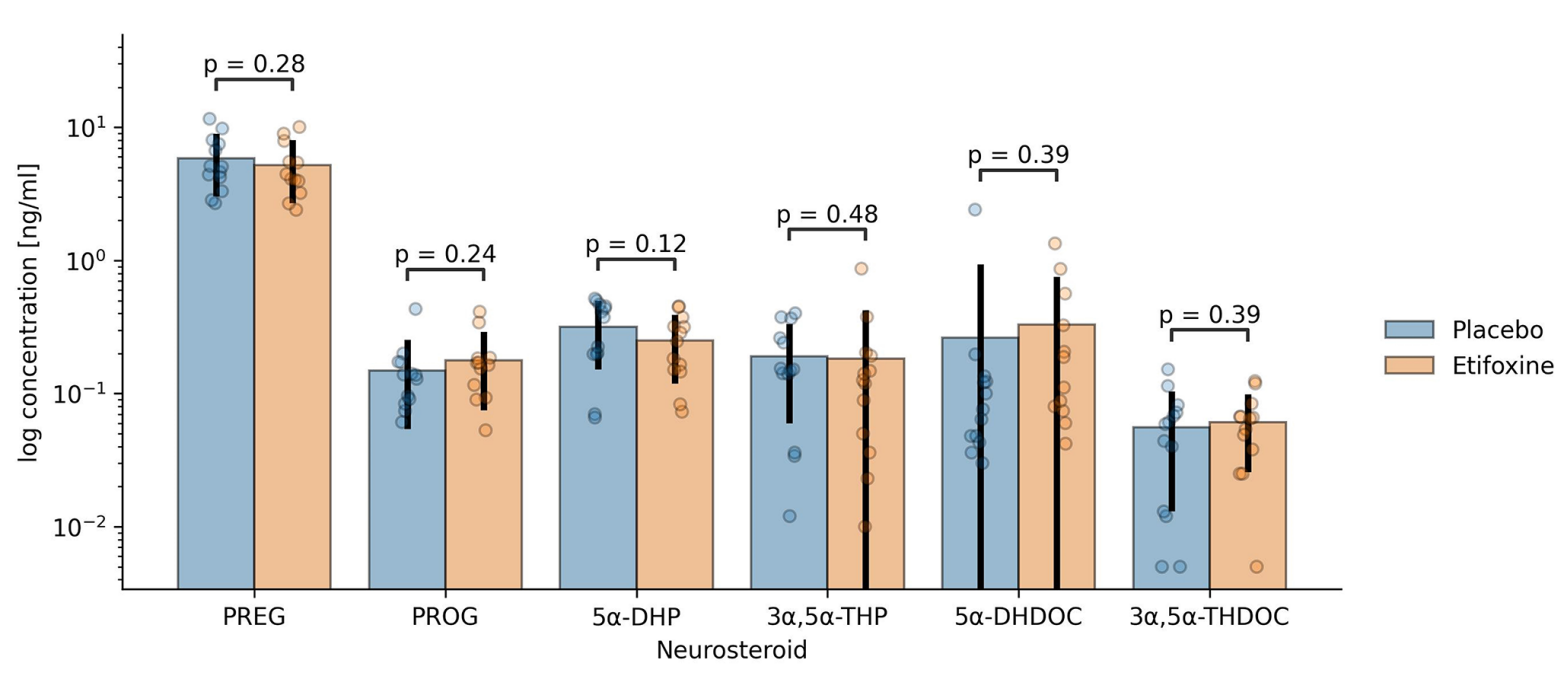
Steroid profile in plasma following administration of etifoxine and placebo in healthy male volunteers. Steroid concentrations in ng/ml are shown logarithmized on the y axis. Error bars indicate 95% confidence intervals. No significant effect of the etifoxine on plasma steroids was detectable. PREG: pregnenolone, PROG: progesterone, 5α-DHP: 5α-dihydrodroprogesterone, 3α,5α-THP: 3α,5α-tetrahydroprogesterone, 5α-DHDOC: 5α-dihydrodeoxycorticosterone, 3α,5α-THDOC: 3α,5α-tetrahydrodeoxycorticosterone

Figure 3 (Fig. 3) shows peripheral plasma levels of pregnenolone (PREG), progesterone (PROG), 5α-dihydroprogesterone (5α-DHP), 3α,5α-tetrahydroprogesterone (3α,5α-THP, allopregnanolone), 5α-dihydrodeoxycorticosterone (5α-DHDOC), and 3α,5α-tetrahydrodeoxycorticosterone (3α,5α-THDOC) on a logarithmic scale (absolute values are provided in Table 1).

**Table 1:** Steroid profile in plasma following administration of etifoxine and placebo in healthy male volunteers. Mean and standard deviation of steroid concentrations in ng/ml. PREG: pregnenolone, PROG: progesterone, 5α-DHP: 5α-dihydrodroprogesterone, 3α,5α-THP: 3α,5α-tetrahydroprogesterone, 5α-DHDOC: 5α-dihydrodeoxycorticosterone, 3α,5α-THDOC: 3α,5α-tetrahydrodeoxycorticosterone

neurosteroid	medication	mean [ng/ml]	standard deviation
PREG	Etifoxine	5.222	2.486
	Placebo	5.832	2.730
PROG	Etifoxine	0.178	0.103
	Placebo	0.148	0.095
5α-DHP	Etifoxine	0.249	0.128
	Placebo	0.317	0.162
3α,5α-THP	Etifoxine	0.183	0.227
	Placebo	0.190	0.131
5α-DHDOC	Etifoxine	0.328	0.403
	Placebo	0.264	0.646
3α,5α-THDOC	Etifoxine	0.061	0.035
	Placebo	0.056	0.044

In this clinical study, no effects of etifoxine were detected in the plasma of male volunteers. This is in contrast to findings in rats, where etifoxine caused increases in progesterone and 3α-reduced steroids in both brain and plasma [83, 84] as well as in cellular systems [56]. Presumed differences in tissue-specific enzymatic machinery and between rats and humans preclude further conclusions. A part of the steroid pool, like allopregnanolone, is mainly formed in peripheral glands [85], whereas neuronal tissue provides enzymes such as the 5α-reductase and 3α-hydroxysteroid dehydrogenase to synthesize meaningful local concentrations of neurosteroids [85–87]. In rats, plasma levels are not fully correlated and much lower compared to their levels in neural tissue [88, 89]. Although these findings seem to weaken the role of peripheral plasma levels of neurosteroids as sensitive clinical biomarkers, measurable differences in plasma levels of specific neurosteroids could still be an important marker of pathological conditions with or without reflecting localized brain processes [90]. Ongoing clinical research in our group aims to study the sensitivity of peripheral steroid levels to therapeutic manipulation, e.g. by etifoxine treatment under pathological conditions [91].

### Etifoxine and GABAkines in the treatment of depression

Because TSPO ligands may induce neurosteroidogenesis, they may represent an alternative approach to exogenously applied 3α-reduced steroids such as brexanolone or zuranolone in the treatment of postpartum depression or major depressive disorder. As outlined above, our group is currently performing a first proof of concept study assessing the potential of etifoxine as adjunct treatment in depression in relation to neuroimaging and microbiome parameters. Moreover, modifications of the etifoxine molecule, such as the GABAkine GRX-917 [92], may provide TSPO ligands with improved pharmacokinetic properties. It is noteworthy that side effects such as sedation, tolerance development and abuse liability, which are typical for benzodiazepines, have not been reported for TSPO ligands so far, which further supports their investigation as a novel treatment option.

Conclusion and outlook.

The neurosteroid field is known for decades. However, it has recently gained considerable interest due to industrial efforts, e.g., by SAGE/Biogen, to develop 3α-reduced steroids such as brexanolone and zuranolone as novel therapeutic agents for affective disorders. With respect to postpartum depression, it is quite clear that the dramatic drop in progesterone after childbirth leads to a corresponding drop in 3α-reduced steroids, which may contribute to the psychopathology of postpartum depression. As a clinical consequence, the administration of exogenous 3α-reduced steroids has a solid pathophysiological rationale. In fact, the portfolio of studies presented has led to the approval of brexanolone and zuranolone for the treatment of postpartum depression. The situation is less clear in major depressive disorder. Although subtle changes in neurosteroid composition have been reported in major depressive disorder and anxiety disorders, and antidepressants have been shown to interfere with neurosteroidogenic enzymes, the magnitude of alterations is only marginal compared to postpartum depression. Nevertheless, it is intriguing that the study portfolio presented by SAGE/Biogen suggests a rapid antidepressant potential of zuranolone also in major depressive disorder. However, zuranolone has not been approved by the FDA for major depressive disorder due to several concerns that need to be addressed. According to the authors, the following questions should be addressed: What will be the side-effect profile after a more prolonged administration beyond 14 days? Will there be abuse liability or tolerance effects? What will be the efficacy over a longer period of time? Will these agents find their place as a monotherapy or as adjuncts to standard antidepressants? Will interval therapy become a novel treatment regimen? Moreover, the induction of endogenous neurosteroidogenesis via TSPO ligands such as etifoxine or derivatives may be an interesting alternative approach. Will these two approaches provide similar clinical effects, or will there be a different clinical profile given the broad spectrum of action of TSPO ligands? In conclusion, it is encouraging that the progress made in the field of neurosteroids and TSPO has now opened the door to new treatment options. Hopefully, these will find their way as alternative strategies to existing pharmacological and non-pharmacological treatments for affective disorders.
